# Voyages in Vacation,—V

**Published:** 1888-10-27

**Authors:** 


### VOYAGES IN VACATION.—V.

### BY ONE IN SEARCH OF RESTFUL RESTORATIVES.

*(Continued from page 44.)*

### V.—COMPANIONS AND ENTERTAINMENTS.

WE have said that pleasant companions are essential to the full enjoyment of a voyage of any duration. This is so palpable a fact that few will venture to gainsay it. There are, however, certain confirmed old bachelors, and ladies of a certain or uncertain age, who declare their reason for taking such a cruise to be an absorbing desire to enjoy their own society, and to escape from their friends and acquaintances. All pleasure yachts and most passenger ships will be sure to carry a few such individuals regularly. Another class join a vessel to enable them to indulge their appetites or idiosyncrasies without let or hindrance. A morphiamaniac, or one who indulges in liberal potations, may conclude that so long as he keeps quiet and pays his way no one will interfere with his habits or proceedings. This is largely so, but the confirmed tippler is not usually allowed to remain on board, and when once his proclivities are indulged in, he is soon put ashore. The conditions under which passengers are booked give the owners great latitude in this respect, which they do not hesitate to avail themselves of when occasion warrants it. The quiet soaker is a different and more difficult subject to handle, and as freedom of action must be given to the passengers, the owners cannot well interfere unless the comfort of others is concerned. It once happened, we believe, that two adjacent cabins were occupied on the same voyage by two men of totally diverse dispositions. One was a toper par excellance, the other sober to a degree bordering on harmful self denial. The first man knew the second had a bottle of brandy in his cabin which he never touched, and so he determined to have at any rate a share of it. The toper accordingly entered his neighbour's state room one night after bedtime, felt about for the glass, and finding something in it, without more ado threw the contents out of the port-hole. When the abstemious one got up in the morning, he not only found his brandy had been tapped, but he also missed his false teeth, which he had set to soak in the tumbler before getting into bed. His distress knew no bounds, but he never realised the cool effrontery of his midnight visitor, nor did he connect the stolen brandy with the missing teeth.

Of course anyone who goes on a voyage for pleasure may reasonably expect to find a few passengers who will afford him congenial companionship. Still, the experienced traveller will plan his sea trip in advance, so as to enable him to make up a party sufficiently large to be self-contained, and so constituted as to secure a leaven of qualities likely to prove helpful and advantageous throughout the cruise. Youth is the time for sound health, vigorous spirits, and keen enjoyment; therefore the presence of young people on pleasure yachts is a *sine quâ non* to the perfect happiness of everybody. So much is this the case that owners would be well advised to reduce their terms a little for young people of both sexes, whose means are not usually equal to those of their elders, and who might thus be tempted to join many ocean-going parties. Musical and dramatic as well as social qualities are, of course, at a premium on a steamer, and to be blessed with one or more gifts of this character is to place one in a position of great popularity. Of course, it is not possible to prevent the shipment of the invalid, the hypochondriac, or the hysterical, and a specimen of all three varieties is pretty certain to be encountered. There will, however, be small objection to their presence if they are unobtrusive and quiet. But if the poor invalid has a loud, hacking cough at night, should the hypochondriac be obtrusively persistent in thrusting his presence upon his fellows by objecting to music or any form of general amusement which creates innocent fun and enjoyment, or if the dear hysterical creature makes herself a nuisance by eternally reciting her ailments, real or supposed, then such goods are altogether out of place amongst a cargo of passengers whose first, main, and legitimate objects are pleasure, relaxation, and enjoyment. It would be a good thing for the success of these cruises if the owners could arrange for every person to call at the office before their passage money is accepted, and if they would boldly say on certain voyages "no invalids or 'cranks' will be allowed to book berths for this cruise." Still, the only true way to secure pleasant companions and the maximum of fun is, no doubt, to make up a party of friends, who can then, if necessary, amuse each other and have a good time, whatever else may happen to others less fortunately circumstanced and less prudently thoughtful.

The enjoyment and benefits to be derived from a pleasure cruise such as we are considering must be largely influenced by the personnel of the ship's officers. If the captain, besides being a good seaman, is also a man of the world and a gentleman, then the passengers' comfort will be thereby materially increased. Some captains hold to the view, and act upon it, that if they navigate the ship and occasionally lift their hat to a lady passenger with whom they may exchange a few commonplaces on rare occasions, they are carrying out the wisest policy to secure their own happiness and that of those who sail under their command. This plan of leaving the passengers severely alone is all very well for the captain, but it is fatal to the real success of any cruise where passengers are carried. If the captain will interest himself in the passengers, see that everybody is happy, study the peculiarities of each, and set himself, by the exercise of tact, to harmonise differences and to secure that any talent is encouraged to display itself for the general enjoyment, he will soon find himself the commander of the most popular passenger-carrying vessel afloat. It is an excellent practice where the cruise is to last three weeks or more, for the captain to confer with the passengers at the outset, so that a small committee may be organised, and arrangements made to provide regular amusements throughout the voyage. In this way time will be saved, the utmost efficiency and comfort secured, and general satisfaction created. The doctor on such vessels will be voted a *persona grata* or a fool. If he displays the qualities, and possesses the manners and trained diplomacy of a physician, he will be a hero and a blessing. If, on the other hand, he is an uninteresting and raw recruit, without tact, presence, energy or manner, he will find his life a burden to himself, and will be voted a nuisance by the passengers. The doctor can do much to promote the good temper and enjoyment of all on board, and will so be of very material service to the owners of a pleasure yacht. For this reason, and for one we shall consider later, he should be specially selected and paid by the owners, who, if they have discretion, and an eye to their own interests, will not change this officer every cruise to save a few pounds in salary, but will take trouble to find the right man as doctor, and when they have found him, to retain his services for a whole season at the very least. A ship's surgeon, for example, who, from laziness or indifference, does not go round to visit, in a friendly way, all those who are sick when the sea is rough and disturbing, is not worth his salt, and is certainly quite out of place on a pleasure yacht.

In the same way it is desirable that the crew, stewards, and officers of these pleasure yachts should be engaged for the whole season, and not merely for the cruise. Upon the stewards will devolve the duty of giving concerts, nigger and other entertainments at regular intervals. This class of amusement can of course only be provided with success where the crew and stewards are retained for the season and not merely for the cruise. The excellence of these musical diversions must largely depend upon the interest taken in such matters by the chief officers. Where the ship's surgeon or the captain co-operate with the purser and stewards, and give them every encouragement and assistance, there the entertainments will be excellent. Where this is not the case, success can hardly be expected, and if attained it will reflect special honour upon the purser. On the *Victoria*, Mr. Haines, the courteous and obliging purser, possesses histrionic abilities of a high class, and he is thus a very host in himself at an evening symposium. There is no reason why the ship's company of a pleasure yacht should not be specially selected with the view of securing a full cargo every voyage. Passengers are about as profitable a cargo as any vessel can well carry, and for this reason the wise selection of officers and crew, by promoting an increasing and sustained demand for accommodation, must secure the largest possible return on the capital invested by the owners.

*(To be continued.)*

